# Inositol-Requiring Enzyme 1-Mediated Downregulation of MicroRNA (miR)-146a and miR-155 in Primary Dermal Fibroblasts across Three *TNFRSF1A* Mutations Results in Hyperresponsiveness to Lipopolysaccharide

**DOI:** 10.3389/fimmu.2018.00173

**Published:** 2018-02-06

**Authors:** Stephanie R. Harrison, Thomas Scambler, Lylia Oubussad, Chi Wong, Miriam Wittmann, Michael F. McDermott, Sinisa Savic

**Affiliations:** ^1^Leeds Institute of Rheumatic and Musculoskeletal Medicine (LIRMM), Leeds, United Kingdom; ^2^Centre for Skin Sciences, Faculty of Life Sciences, University of Bradford, Bradford, United Kingdom; ^3^National Institute for Health Research–Leeds Biomedical Research Centre, Leeds, United Kingdom; ^4^Department of Clinical Immunology and Allergy, St James’s University Hospital, Leeds, United Kingdom

**Keywords:** tumor necrosis factor-receptor-associated periodic syndrome, unfolded protein response, inisitol-requiring enzyme 1, lipopolysaccharide, toll-like receptor 4, microRNA-146a, microRNA-155, autoinflammation

## Abstract

Tumor necrosis factor (TNF)-receptor-associated periodic fever syndrome (TRAPS) is a rare monogenic autoinflammatory disorder characterized by mutations in the *TNFRSF1A* gene, causing TNF-receptor 1 (TNFR1) misfolding, increased cellular stress, activation of the unfolded protein response (UPR), and hyperresponsiveness to lipopolysaccharide (LPS). Both microRNA (miR)-146a and miR-155 provide negative feedback for LPS-toll-like receptor 2/4 signaling and cytokine production, through regulation of nuclear factor kappa B (NF-κB). In this study, we hypothesized that proinflammatory cytokine signaling in TRAPS downregulates these two miRs, resulting in LPS-induced hyperresponsiveness in TRAPS dermal fibroblasts (DFs), irrespective of the underlying genetic mutation. Primary DF were isolated from skin biopsies of TRAPS patients and healthy controls (HC). TNFR1 cell surface expression was measured using immunofluorescence. DF were stimulated with LPS, interleukin (IL)-1β, thapsigargin, or TNF, with and without inositol-requiring enzyme 1 (IRE1) inhibitor (4u8C), following which miR-146a and miR-155 expression was measured by RT-qPCR. IL-1β, IL-6, and TNF secretion was measured by enzyme-linked immunosorbent assays, and baseline expression of 384 different miRs was assessed using microfluidics assays. TNFR1 was found to be expressed on the surface of HC DF but expression was deficient in all samples with TRAPS-associated mutations. HC DF showed significant dose-dependent increases in both miR-146a and miR-155 expression levels in response to LPS; however, TRAPS DF failed to upregulate either miR-146a or miR-155 under the same conditions. This lack of miR-146a and miR-155 upregulation was associated with increased proinflammatory cytokine production in TRAPS DF in response to LPS challenge, which was abrogated by 4u8C. Incubation of HC DF with IL-1β led to downregulation of miR-146a and miR-155 expression, which was dependent on IRE1 enzyme. We observed global dysregulation of hundreds of other miRs at baseline in the TRAPS DF. In summary, these data suggest a mechanism whereby IL-1β, produced in response to activation of the UPR in TRAPS DF, downregulates miR-146a and miR-155, by inducing IRE1-dependent cleavage of both these miRs, thereby impairing negative regulation of NF-κB and increasing proinflammatory cytokine production.

## Introduction

Tumor necrosis factor (TNF)-receptor-associated periodic fever syndrome (TRAPS) is a rare monogenic autoinflammatory disorder characterized by recurrent attacks of fever and inflammation, predominantly affecting fibroblast-rich tissues such as serosal surfaces, synovial joints, and the deep dermis of the skin (1). The disease is both clinically and genetically heterogeneous, with over 140 mutations reported to date (2). Likewise, a number of molecular mechanisms have been implicated in the pathogenesis of TRAPS, including abnormal TNF-receptor 1 (TNFR1) receptor cleavage (3), ligand-independent activation of mutant TNFR1 (4–6), activation of nuclear factor kappa B (NF-κB)/mitogen-activated protein kinase (6–10), generation of mitochondrial reactive oxygen species (7, 11), and TNFR1 misfolding and retention within the endoplasmic reticulum (ER), leading to activation of the unfolded protein response (UPR) (7). This latter cellular stress response appears to be limited to selective activation of the ER-associated endonuclease, inositol-requiring enzyme 1 (IRE1) (8, 12); however, this activation can have significant proinflammatory effects as shown by Martinon et al. They demonstrated that lipopolysaccharide (LPS) can activate IRE1, *via* the TLR4 signaling pathway. The spliced X-box binding protein 1 (XBP1) transcription factor, generated *via* activation of IRE1, can subsequently bind to the promoter regions of TNF and interleukin (IL)-6 (13). In turn, we have shown that inflammatory cytokines, such as TNF, can also activate the IRE1 arm of the UPR resulting in synthesis of XBP1s (14). Therefore, in TRAPS patients, the coexistence of low-level ER stress, with resultant local production of proinflammatory cytokines, can promote chronic activation of IRE1, and subsequent heightened responsiveness of TRAPS cells. These findings corroborate the observations that TRAPS cells are hyperresponsiveness to low-dose LPS, with increased production of proinflammatory cytokines, the release of which leads to clinical manifestations persisting for periods of weeks to months (1, 15, 16).

The traditional role for IRE1, as part of the UPR, relates to the endonuclease function of this enzyme and its ability to target a variety of mRNA and microRNA (miRs) species, and, in this way, limit protein production and help to resolve ER stress (17). IRE1 regulates the expression of mRNA, and miRs *via* regulated IRE1-dependent decay (RIDD) (18). In this way, IRE1 controls protein exit from the ER, including the levels of proteins that go on to be involved in regulation of ER processes, at both the genetic and epigenetic level. Control of miRs can lead to significant modulation in activity of cellular pathways by determining either cell survival or death (19). miRs, which are small non-coding RNAs that regulate mRNA expression by translational inhibition (20), usually have multiple targets, which may be found on the same and/or different molecular pathways (20).

The co-expression of miR-155 and miR-146 in human monocytes, in response to LPS, was first shown in 2006 (21). Despite evidence suggesting both pro- and anti-inflammatory actions for miR-155, in different contexts, numerous publications have demonstrated that both miR-155 and -146a target a number of downstream signaling pathways involved in toll-like receptor 4 (TLR4)-mediated LPS responses (22, 23), suggesting that, collectively, these miRs regulate a negative-feedback loop to prevent excessive TLR4 activation. In 2011, Schulte et al. suggested a more refined role for these two miRs (24); they used a graded LPS challenge to show that miR-146a was necessary for prevention of TLR4 responses, at sub-inflammatory doses of LPS, which might be relevant to maintaining tolerance to the host’s own microbiome. On the other hand, miR-155 was found to limit TLR4 responses following exposure to higher, proinflammatory doses of LPS. Thus, failure to upregulate these miRs may lead to chronic hyperresponsiveness of the TLR4 pathway.

We therefore hypothesized that the intracellular levels of miR-155 and miR-146a may be reduced in TRAPS cells, possibly due to targeted destruction by IRE1. Furthermore, the proinflammatory milieu of TRAPS cells, particularly due to paracrine effects of TNF and IL-1β, would facilitate this process. We decided to focus on the effects of IL-1β, since this cytokine appears to be critical to the disease pathogenesis and clinical manifestations of TRAPS, and also because anti-IL-1 therapy is now the treatment of choice for this condition (25). Furthermore, we elected to study these effects in dermal fibroblast (DF) from three TRAPS patients, who harbored three different *TNFRSF1A* mutations, since many of the clinical manifestations of TRAPS are localized to fibroblast-rich tissues, and we wanted to show that this is not a mutation-specific phenomenon.

## Materials and Methods

### Patients and Cells

Primary DF were obtained by digestion of skin biopsies from patients with three different TRAPS mutations; T50M and C88R missense mutations, and a C158delinsYERSSPEAKPSPHPRG (c.472 + 1 G > A) splice site mutation in the *TNFRSF1A* gene. Primary DF from six healthy controls were used as controls. Healthy and patient derived DF were cultured and passaged in the same way, using Dulbecco’s Modified Eagle Medium [Gibco (Life Technologies), Paisley, UK] supplemented with fetal bovine serum (10%v/v), penicillin–streptomycin (1%v/v), and minimum essential medium non-essential amino acids (1% v/v) or Fibroblast Cellutions media (Cambridge Biosciences, Cambridge, UK). All cells had a morphology and growth pattern consistent with fibroblast cells and expressed fibroblast-specific protein.

### Immunofluorescence

Fibroblasts were grown on culture chamber slides (BD Biosciences, Bedford, MA, USA). Adherent cells were fixed using 4% paraformaldehyde, at 37°C for 30 min and blocked using 2% bovine serum albumin or MACS buffer (Miltenyi Biotec, Surrey, UK) for 30 min at room temperature (RT, 21°C). Slides were then incubated with 10 ug/μl of TLR4 and TNFR1 primary antibodies (R&D systems, Abingdon, UK) and goat IgG isotype controls (gift from Dr. Stephenson) for 1 h. Slides were washed three times using 1% phosphate-buffered saline, and then incubated with equivalent concentrations of secondary antibody [FITC polycloncal rabbit anti-goat (Abcam, Nottingham, UK)] for 1 h. Slides were then washed (as described previously). Coverslips were mounted, using ProLong Diamond anti-fade mountant containing DAPI, and visualized using the Carl Zeiss Imager Z1 immunofluorescence microscope. Images were captured using the AxioCam MRc5 camera and AxioVision Rel software, v4.7.

### Cell Stimulations with LPS, IL-1β, IL-6, and TNF

For all experiments, a total of 1.2 × 10^5^ cells were seeded in 12-well culture plates and allowed to adhere overnight. Cells were then stimulated with 10.0, 1.0, and 0.1 ng/mL LPS for 6 h (HC, *n* = 6; TRAPS, *n* = 3). In separate experiments, the HC DF were incubated with IL-1β (10 ng/mL), TNF (10 ng/mL), and thapsigargin (Tg) (300 nM) with or without the IRE1 inhibitor 4μ8C (50 µM), for 4 h. Supernatants were collected and stored at −80°C for analysis of cytokine secretions. RNA was extracted from cells using TRIzol reagent.

### RNA Extraction

RNA extraction and in-column DNAse treatment was performed, using DirectZOL RNA Miniprep Kit (Zymo Research, Irvine, CA, USA) or PureLink RNA Mini Kit (Ambion), according to the manufacturers’ protocol.

### TaqMan Low-Density Array

RNA-derived cDNA from unstimulated cells was synthesized using MegaPlex RT Human Pool A v3.0. Expression of 380 microRNAs was then quantified using TaqMan Low-Density Array Card A. Fold change of expression of individual genes was calculated using the ΔΔ*Ct* method, normalizing to the housekeeping gene U6, and unsupervised hierarchical clustering analysis was performed, using Cluster v3.0 and Java Tree View. In addition, all miRs increased/decreased more than threefold compared with HC were analyzed, using the DIANA miRpath bioinformatics platform to identify pathways predicted to be regulated by miRs.

### TaqMan miR-146a and miR-155 Assays

RNA-derived cDNA was synthesized from 100 ng total RNA, using TaqMan miRNA Reverse Transcription Kit (Applied Biosystems) according to the manufacturers’ protocol. Individual qPCR reactions were performed in duplicate, using MicroAmp Optical 96-well reaction plates and the ABI 7500 thermal cycler (Applied Biosystems). Expression of the target genes was determined by the ΔΔ*Ct* relative quantification method, normalizing to the housekeeping gene, RNU6B.

### IL-1β Enzyme-Linked Immunosorbent Assays (ELISA)

Interleukin-1β ELISA (Novex, Life Technologies) were used to measure levels of the proinflammatory cytokine, IL-1β, in cell culture supernatant samples, according to manufacturers’ protocol. Absorbance was read at 450 nm (with 570 nm γ-correction) using Mirthas LB940 (Berthold Technologies, Bad Wildbad, Germany) and MikroWin 2000 analysis software.

### Statistical Analyses

Details of different statistical methods and software packages used are provided under the relevant section headings above. For some experiments, due to scarcity of samples, analyses for each TRAPS mutation represent the results of a single individual, and, therefore, formal tests of statistical significance were not significant but do imply trends. Statistical significance was determined using a two-way ANOVA Sidak’s multiple comparisons test, as indicated in the relevant figure legends.

## Results

### TRAPS DF Have Normal Cell Surface Expression of TLR4 but Reduced Expression of TNFR1

**Figure 1:**
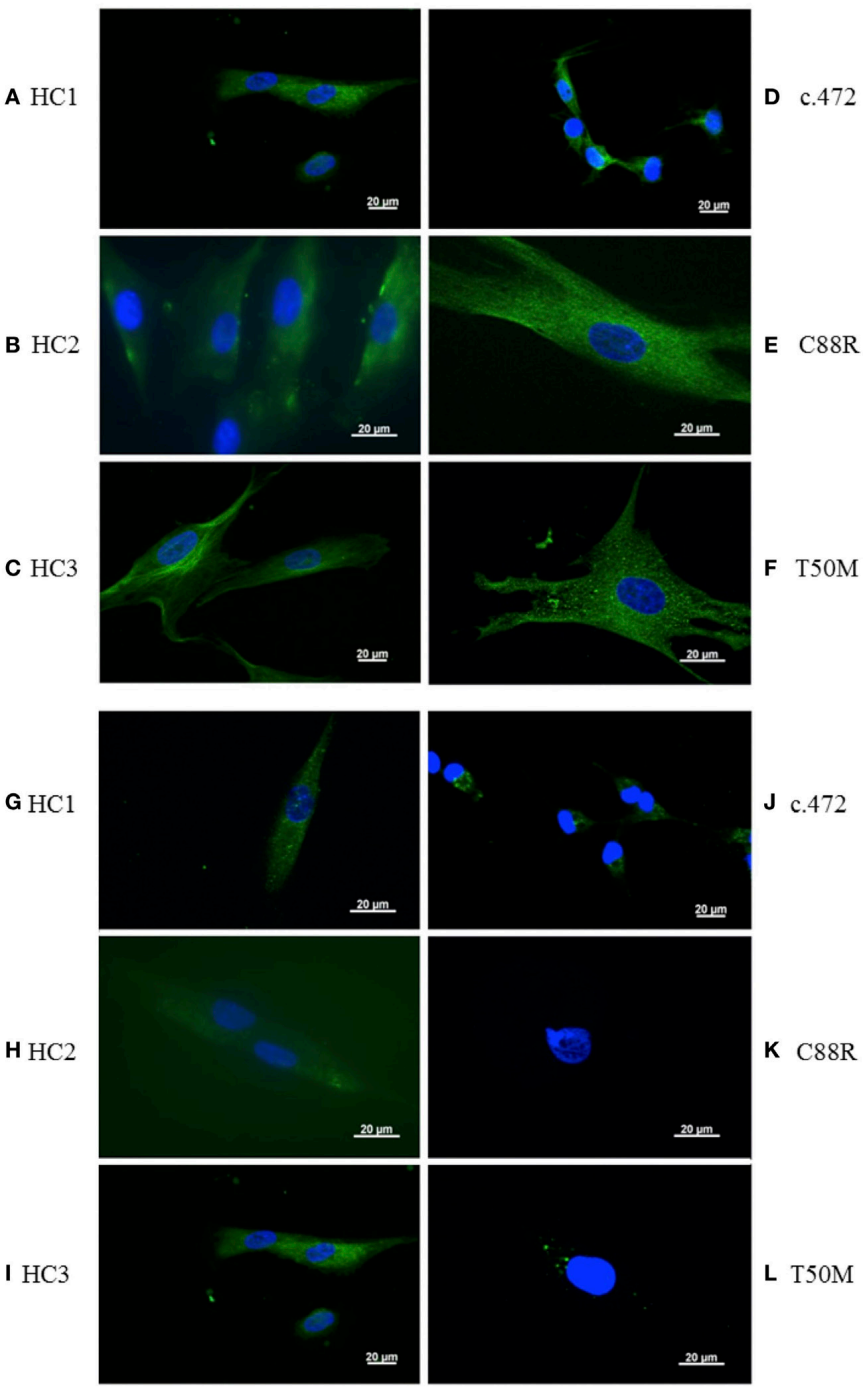
Detection of surface toll-like receptor 4 (TLR4) and tumor necrosis factor (TNF)-receptor 1 (TNFR1) on healthy control (HC) and TNF receptor-associated periodic fever syndrome (TRAPS) dermal fibroblast (DF) using immunofluorescence. Expression of TLR4 (green) from TRAPS (*n* = 3) and HC (*n* = 3) DF **(A–F)**. Expression of TNFR1 (green) in TRAPS (*n* = 3) and HC (*n* = 3) DF **(G–L)**. DAPI (blue) is used to stain the nucleus. Isotype and no primary antibody controls were all negative (data not shown). Each image is representative of three images of one to five cells from a slide of 5 × 10^3^ cells from each TRAPS mutation and three HCs.

Healthy human DF regulate inflammatory responses by expressing various cell surface receptors, including TLR4 (25); therefore, to determine if TRAPS DF also continue to express TLR4 *in vitro*, and successfully respond to LPS stimulation, we studied the expression of TLR4 on the cell surface in primary DF, from HC (*n* = 3) and also from TRAPS patients, respectively, bearing T50M, C88R, and c.472 + 1 slice site *TNFRSF1A* mutation. Surface expression of TLR4 in all three TRAPS DF was comparable with HC (Figures 1A–F).

We, and others, have previously shown that the mutated TNFR1 species tend to aggregate within the cells and to express less TNFR1 on the cell surface (7, 26). To date, only one study has reported on TNFR1 expression in primary DF, from TRAPS patients bearing the C33Y mutation, which found reduced levels of sTNFR1 and also reduced receptor shedding when compared with HC (27, 28). Using immunofluorescence, we also demonstrated that TNFR1 expression was markedly reduced/absent in all DF from the three TRAPS patients compared with cells from HC (Figures 1G–L).

### Negative Regulation of LPS Signaling by miR-146a and miR-155 Is Defective in TRAPS DF

**Figure 2:**
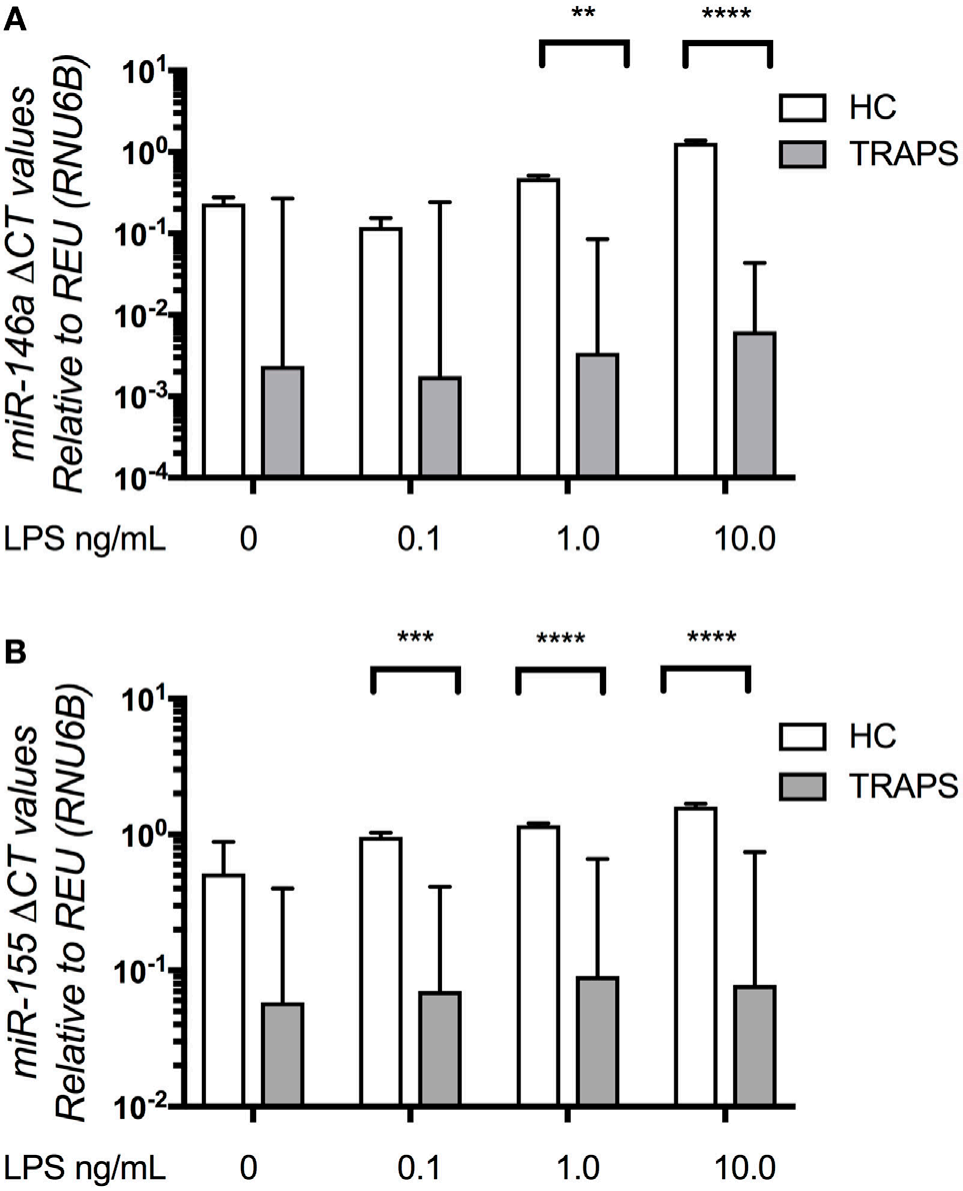
MicroRNA (miR)-146a, miR-155 in tumor necrosis factor (TNF) receptor-associated periodic fever syndrome (TRAPS) and healthy control (HC) dermal fibroblast (DF) following graded lipopolysaccharide (LPS) challenge. TRAPS (*n* = 3) and HC (*n* = 4) DF miR-146a and miR-155 RNA expression **(A,B)** measured by Taqman qPCR. Data were converted to a logarithmic scale base 10. The two-way ANOVA (*p* = ≤ 0.05) test was used to determine statistical significance.

miR-146a and miR-155 have been shown to operate at different levels of LPS stimulation. miR-146a is active at sub-inflammatory doses of LPS, thus regulating the threshold of activation, whilst miR-155 acts as a “molecular brake” of proinflammatory gene expression, once the miR-146a-dependent barrier to LPS-triggered inflammation has been breached (24). Thus, we used a graded LPS challenge (0.1–10.0 ng/L) to study these miR responses in DF from TRAPS patients and HC. Neither miR-146a nor miR-155 levels were increased in TRAPS DF, following the graded challenge, even after adding 10 ng/mL of LPS for 6 h (Figures 2A,B). This was a consistent observation for all three TRAPS mutations. In contrast, HC DF showed a dose-dependent increase in both miR-146a and miR-155 (Figures 2A,B). As expected, IL-1β levels were significantly higher in DF from TRAPS patients challenged with graded LPS (Figure S1 in Supplementary Material).

### Pre-Treatment with Proinflammatory Cytokines Promotes miRNA Dysregulation in HC DF

In addition to localized connective tissue manifestations, TRAPS is also characterized by systemic inflammatory responses, due to the release of the proinflammatory cytokines (1, 9, 29). Furthermore, previous work, by our group and others, has reported UPR activation in TRAPS, associated with increased production of IL-1β (1, 7, 13). Therefore, we postulated that miR-155 and miR-146a expression in TRAPS DF may be suppressed by IL-1β. To test this hypothesis, HC DF cells (*n* = 4) were incubated with IL-1β, TNF or Tg, which induces ER stress, IRE1 activation, and UPR. In addition, the IRE1 inhibitor, 4μ8C, was added with Tg and levels of miR-146a, miR-155 were measured.

**Figure 3:**
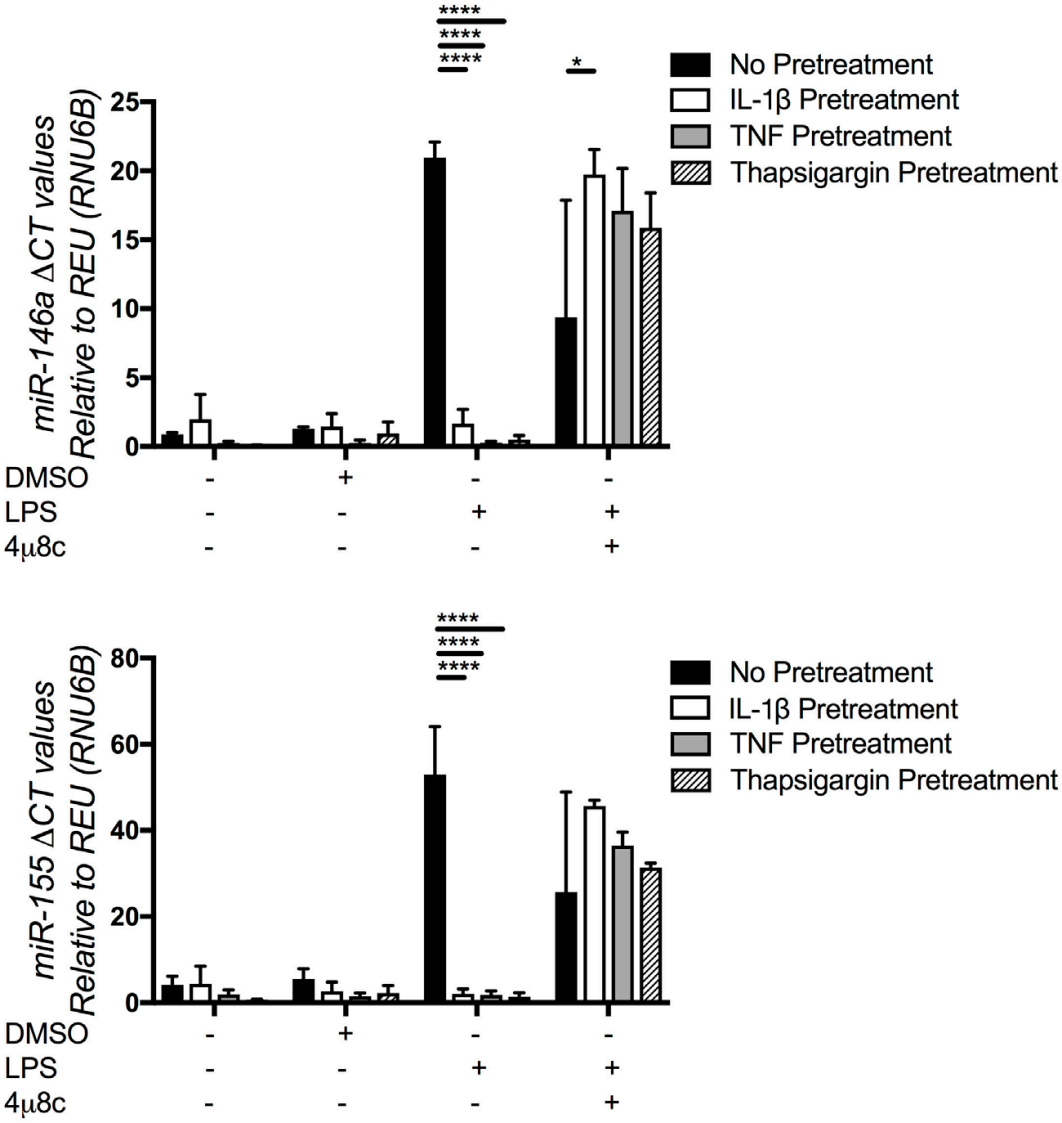
MicroRNA (miR)-146a and miR-155 expression in healthy control (HC) pre-treated with interleukin (IL)-1β, tumor necrosis factor (TNF), and thapsigargin (Tg). Pre-treatment of HC dermal fibroblasts (*n* = 4) with IL-1β (10 ng/mL), TNF (10 ng/mL), and Tg (300 nM). Expression of miR-146a (upper graph) and miR-155 (lower graph) was measured by Taqman qPCR, with and without the addition of the inositol-requiring enzyme 1 inhibitor 4u8C (50 µM). The two-way ANOVA (*p* = ≤ 0.05) test was used to determine statistical significance.

There were no differences found in baseline expression of miR-146a and miR-155 between treated and untreated HC DF (Figure 3). However, there was a significant reduction of LPS-induced miR-146a and miR-155 expression in HC DF when pre-treated with IL-1β, TNF, or Tg, and the effects of Tg were partially reversed by addition of 4μ8C. These results suggest, as previously demonstrated in the literature, that Tg can activate IRE1, lead to UPR activation and IL-1β production. The same effect could also be achieved by incubating cells with TNF and IL-1β, which are both produced following UPR activation.

### Pre-Treatment with 4u8C Reduces Proinflammatory Cytokine Production in TRAPS DF Stimulated with LPS

**Figure 4:**
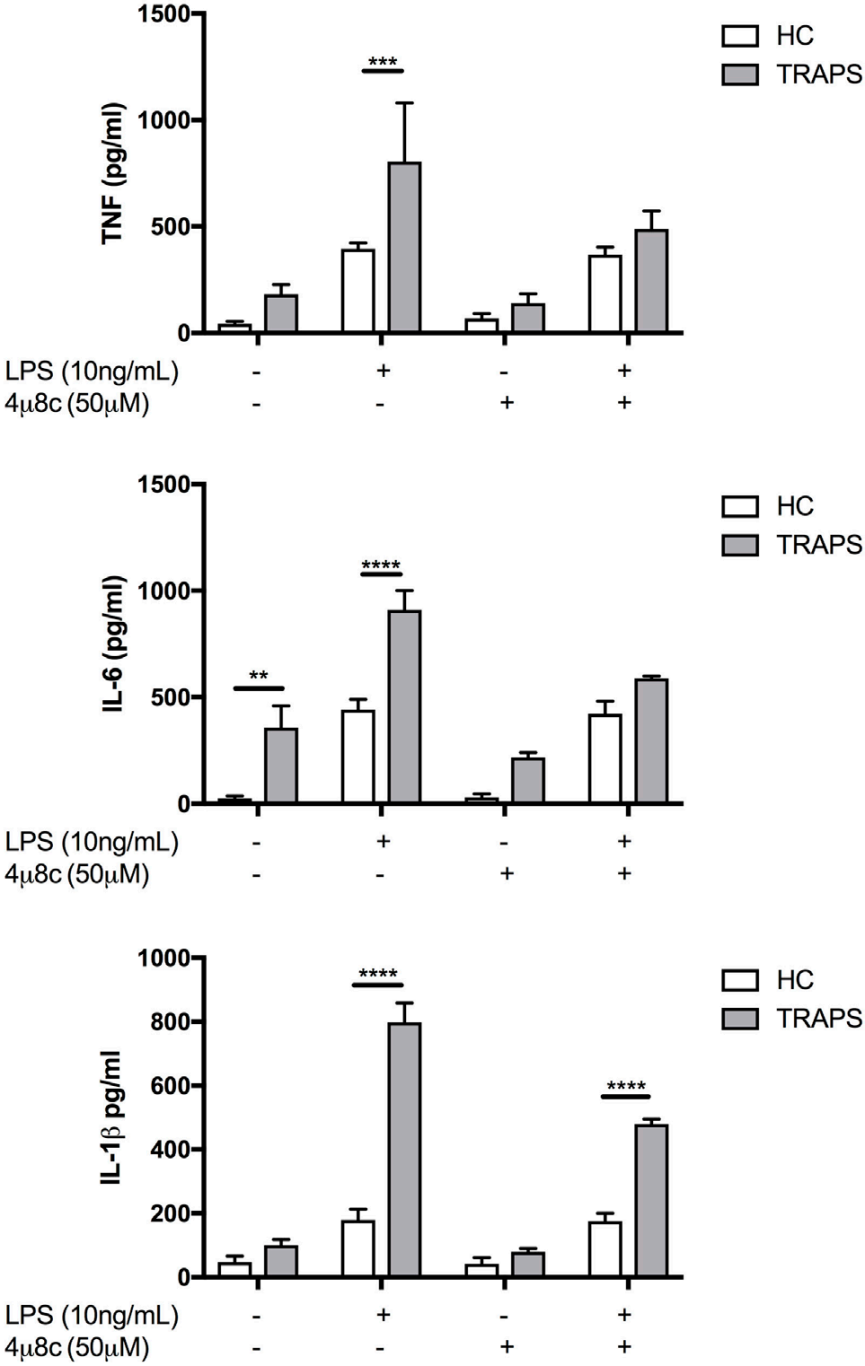
Proinflammatory cytokine production in tumor necrosis factor (TNF) receptor-associated periodic fever syndrome (TRAPS), and healthy control (HC) dermal fibroblast, with and without inositol-requiring enzyme 1 inhibitor 4u8C. Pre-treatment with 4u8C was followed by a stimulation with lipopolysaccharide (LPS) (10 ng/mL) in both TRAPS (*n* = 2) and HC (*n* = 6). The two-way ANOVA (*p* = ≤ 0.05) test was used to determine statistical significance.

To determine whether suppression of the miRs by IL-1β was dependent on IRE1 cleavage, HC and TRAPS cells pre-treated with 4u8C for 4 h before being stimulated with LPS for 4 h. Levels of IL-1β, IL-6, and TNF were increased in HC and significantly higher in T50M and C472 TRAPS DF after LPS stimulation (Figure 4). Following the 4u8C pre-treatment conditions, HC DF proinflammatory cytokine secretion was not altered; however, both TRAPS DF genotypes produced less IL-1β, IL-6, and TNF in response to LPS, with levels that were more like that of the HC DF response. These data are consistent with a model whereby IL-1β downregulation of miR-146a and miR-155 is dependent on IRE1 and leads to increased proinflammatory cytokine production in TRAPs but not HC DF.

### miR Dysregulation Is IRE1 Dependent and Is Present at Baseline in TRAPS DF

**Figure 5:**
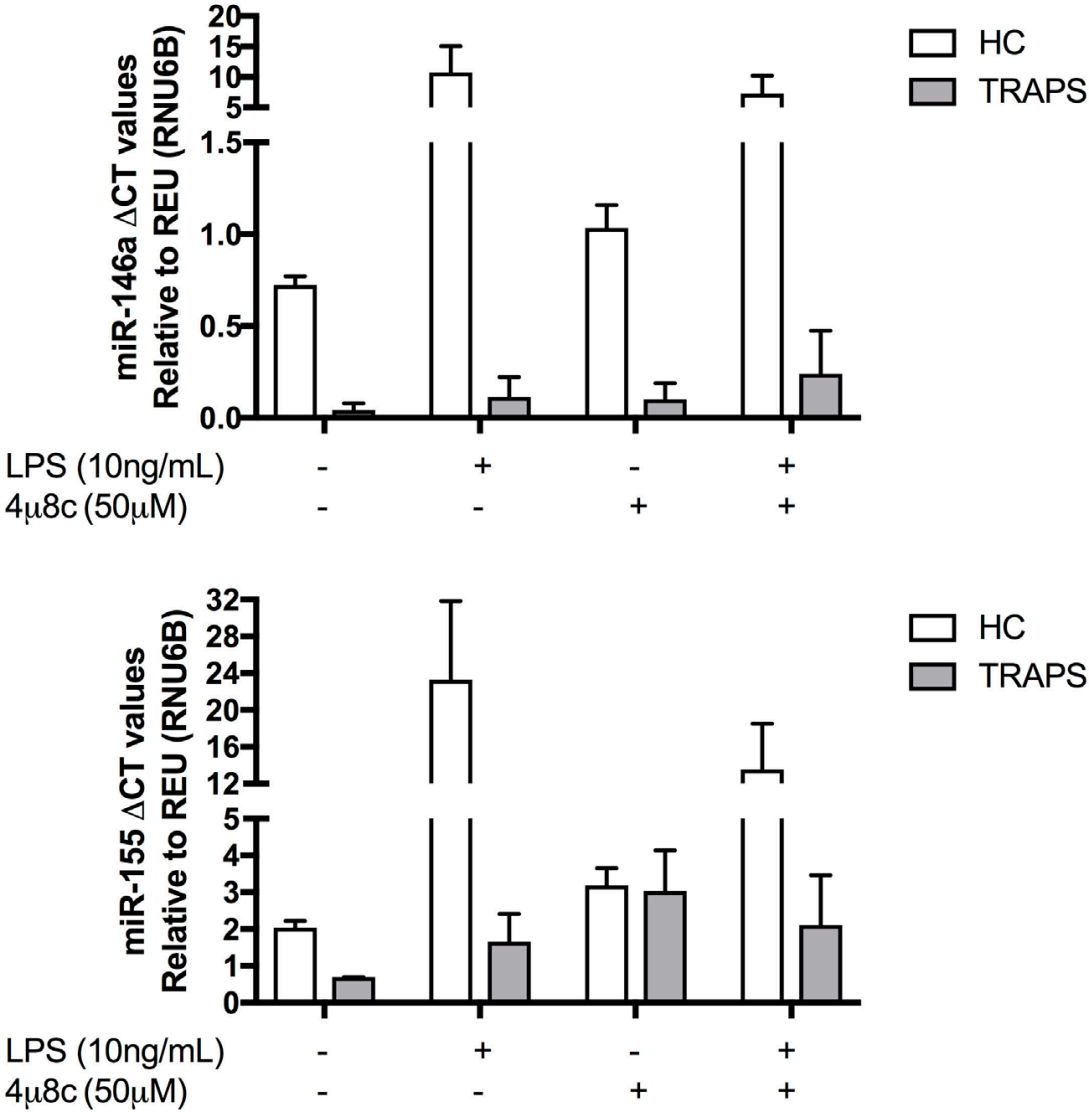
Expression of microRNA (miR)-146a and miR-155 in healthy control (HC) and tumor necrosis factor (TNF) receptor-associated periodic fever syndrome (TRAPS) dermal fibroblast (DF) with and without inositol-requiring enzyme 1 inhibitor 4u8C. Expression levels of miR-146a and miR-155 was measured in TRAPS DF (*n* = 2) compared with HC (*n* = 6). The two-way ANOVA (*p* = ≤ 0.05) test was used to determine statistical significance.

To corroborate the reduced cytokine production seen in response to IRE1 inhibitor 4u8C, both miR-146a and miR-155 were measured under the same conditions (Figure 5). Both miRs are elevated in HC at both baseline and after LPS stimulation, compared with low levels in DF from TRAPS under the same conditions (Figure 5). In both T50M and C472 TRAPS DF, concentrations of both miRs increase after a pre-treatment with IRE1 inhibitor 4u8c. This result suggests that IRE1-dependent degradation of miR-146a and miR-155 not only results in elevated proinflammatory cytokine release (Figure 4) but also can be reversed using small molecule inhibitors.

In addition to miR-146a, and miR-155, a number of other miRs species appear to have unique expression profiles in the sera of patients with TRAPS. Lucherini et al. reported on six miRs that were differentially expressed and, in addition, were able to discriminate TRAPS patients from HC (30). If IRE activation was present in TRAPS DF, which our data suggest is the case, then we would expect other downstream pathways controlled by IRE1, such as RIDD, to also be activated. RIDD has the potential to target other miRs that regulate other cellular and inflammatory processes. Therefore, we investigated whether global expression of miRs in DF might be different between TRAPS patients’ DF and HC. We used TaqMan Low-Density Array gene cards to measure the expression of 384 miRs in DF from all three TRAPS mutations and from three HC at baseline (Figure S2 in Supplementary Material). Full data available from NCBI website accession number GSE109363. Unsupervised hierarchical clustering clearly separated the miR profile of the TRAPS patients from HC, with 11 miRs being differentially expressed ≥2 fold in at least 2 of the 3 TRAPS DF compared with HC. These miRs are implicated in several biological pathways, including ubiquitin-mediated proteolysis, protein processing in the ER, and mRNA surveillance pathways (Table S1 in Supplementary Material). Thus, collectively, our data suggest that miR dysregulation in TRAPS DF activates a number of biological pathways, including mRNA degradation, which could contribute to the hyperresponsiveness to LPS and enhanced proinflammatory cytokine production seen in this condition.

## Discussion

In this study, we show, for the first time, that TRAPS DF with T50M, C472, and C88R mutations in *TNFRSF1A* are hyperresponsive to LPS. We were also able to induce a similar phenotype in healthy DF following stimulation with IL-1β, which was partially reversed using the IRE1 inhibitor 4μ8C. In both cases, IRE1 activation was associated with significantly reduced levels of miR-146a and miR-155 in the cells under study. Finally, we have demonstrated the presence of global miR dysregulation in TRAPS patients, which, alongside the downregulation of miR-146a and miR-155, contributes to the proinflammatory phenotype observed in TRAPS T50M, C472, and C88R genotypes.

Lucherini et al. reported a 6-miR signature to be diagnostic of TRAPS (30); however, to our knowledge, no studies to date have suggested a functional role of miRs in TRAPS pathogenesis. Evidence to date unequivocally points toward important role for miR-146a in numerous inflammatory diseases (22). In the case of miR-155, both pro- and anti-inflammatory functions have been reported (31, 32). The expression of miR-155 does increase in a gradual fashion, similar to most other NF-κB-dependent genes in response to environmental stimuli. This may imply a role for miR-155 in propagating inflammatory responses, particularly since it has been shown to stimulate TNF production by stabilizing its mRNA (33). Furthermore, miR-155 inhibits the translation of the anti-inflammatory molecule, SHIP-1 (31) [reviewed in Ref. (22, 34, 35)]. However, at the same time, miR-155 was shown to suppress some proinflammatory components of the TLR4 pathway, such as TAB 2 and IKKε (33, 36), which suggests a more complex role for this miR in regulation of the inflammatory response. Schulte et al. proposed a scenario whereby, on the one hand, miR-155 may induce negative regulation of autocrine signaling whilst, at the same time, promoting paracrine signaling *via* TNF (24). The end result could activate bystander macrophages, which is essential to deal with infection, but, at the same time, miR-155 may limit the magnitude of the inflammatory response by preventing hyper-activation of the TNF-producing cells. Our data would support such a model. However, to what extent these differential roles of miR-155, or the relationship between miR-146a and miR-155, contribute to specific disease pathogenesis remains to be determined.

Our data also highlight the importance of LPS-hyperresponsiveness in the pathogenesis of TRAPS T50M, C472, and C88R mutations. Our group and others have previously reported this phenomenon in TRAPS, as well as in other inflammatory diseases (37). We have found equivalent levels of surface expression of TLR4 between HC and TRAPS, suggesting that the LPS-hyperresponsiveness observed was not mediated by differences in TLR4 surface expression. Interestingly, TNFR1 knockout mice are resistant to LPS-induced shock, and human monocytes that are deficient in TLR4 are also resistant to LPS (6, 38). Thus, mutant TNFR1 may act in synergy with the TLR4 signaling pathway, triggering increased cellular stress, and consequent lowering of the activation threshold for TLR4-LPS signaling which, in turn, further augments intracellular stress levels in DF with T50M, C472, and C88R mutations in *TNFRSF1A*. In addition, LPS is expressed at the cell surface of gram-negative bacteria, therefore in the LPS-hyperresponsive state, it may be possible for commensal, or subclinical bacterial loads, to trigger an inflammatory response.

Although TRAPS is associated with mutations in *TNFRSF1A* it seems that IL-1β is also critical to the disease pathogenesis. Previous data published by our group, as well as others, have reported high levels of IL-1β in the serum of a number of TRAPS patients (9, 39). Furthermore, anti-IL-1 targeted therapy is now emerging as the treatment of choice for this condition (1, 40–44). Our findings suggest that the role exercised by IL-1β in the pathogenesis of TRAPS is, in part, due to its ability to downregulate miR-146a and miR-155. Furthermore, we propose that the effect could be mediated by the IRE1 branch of the UPR, since the IRE1 inhibitor 4u8C partially reversed inhibition of these miRs. We, and others, have shown that proinflammatory cytokines can induce IRE1 activation; for example, it has been shown that both IL-1β and IL-6 are able to induce ER stress in pancreatic islet cells (45). It is also possible that the endoribonuclease activity of IRE1 may only be required for the initiation of IL-1β production, after which inflammatory cytokine production is driven by the autocrine action of proinflammatory cytokines themselves, particularly given that activation of IRE1 is associated with the induction of apoptosis and cell death (18). For example, IRE1 cleaves TRAF2, activating the JNK signaling pathway, which is important in NF-κB activation and proinflammatory cytokine production (46), and, also, TRAF2 is a recognized target of miR-146a (20). Finally, it has been shown that IRE1 and TNFR1 can combine to form an intracellular molecular complex during cellular stress, leading to UPR activation (47). This plethora of supporting evidence shows that IRE1 plays a significant and complex role in cellular stress responses, in both health and disease.

Thus, in summary, overall our data suggest a model whereby TNFR1 protein misfolding within T50M, C472, and C88R mutations, which previously has been shown to occur in TRAPS cells (7, 8) activates the IRE1 branch of the UPR. IRE1 activation leads to cleavage of miR-146a and miR-155, and consequently results in hyperresponsiveness to LPS and increased proinflammatory cytokine production, such as IL-1β. Subsequently, the paracrine effects of these cytokines as well as the proinflammatory local environment observed in TRAPS maintain the UPR activation state and degradation of miR-146a and miR-155.

## Ethics Statement

All patients provided written informed consent for this Leeds (East) Research Ethics Committee-approved study (REC: 04/Q1206/107).

## Author Contributions

SH, TS, LO, and CW carried out experimental work. SS, MD, and MW provided the patients, designed the study. All contributed toward writing of the paper.

## Conflict of Interest Statement

The authors declare that the research was conducted in the absence of any commercial or financial relationships that could be construed as a potential conflict of interest.

## Supplementary Material

Figure S1 Interleukin (IL)-1β secretion after graded lipopolysaccharide challenge in dermal fibroblast (DF) from healthy control (HC) and patients with tumor necrosis factor (TNF) receptor-associated periodic fever syndrome (TRAPS). Levels of IL-1β secretion observed from TRAPS DF (*n* = 3) compared with HC (*n* = 3). The two-way ANOVA (*p* = ≤ 0.05) test was used to determine statistical significance.

Figure S2 Global expression of microRNAs (miRNAs) in healthy control (HC) and tumor necrosis factor (TNF) receptor-associated periodic fever syndrome (TRAPS). Expression levels of selected miRs observed in TRAPS dermal fibroblast (*n* = 3) compared with HC (*n* = 3). Red indicates higher levels of expression, and green indicates lower levels of expression. A section of the cluster analysis has been magnified to illustrate areas that were clearly different between HC and TRAPS.
