# Supplementary figures and images for: Inositol-Requiring Enzyme 1-Mediated Downregulation of MicroRNA (miR)-146a and miR-155 in Primary Dermal Fibroblasts across Three TNFRSF1A Mutations Results in Hyperresponsiveness to Lipopolysaccharide
Source: Front Immunol. 2018 Feb 6;9:173. doi: 10.3389/fimmu.2018.00173 (PMC5808292; doi:10.3389/fimmu.2018.00173)

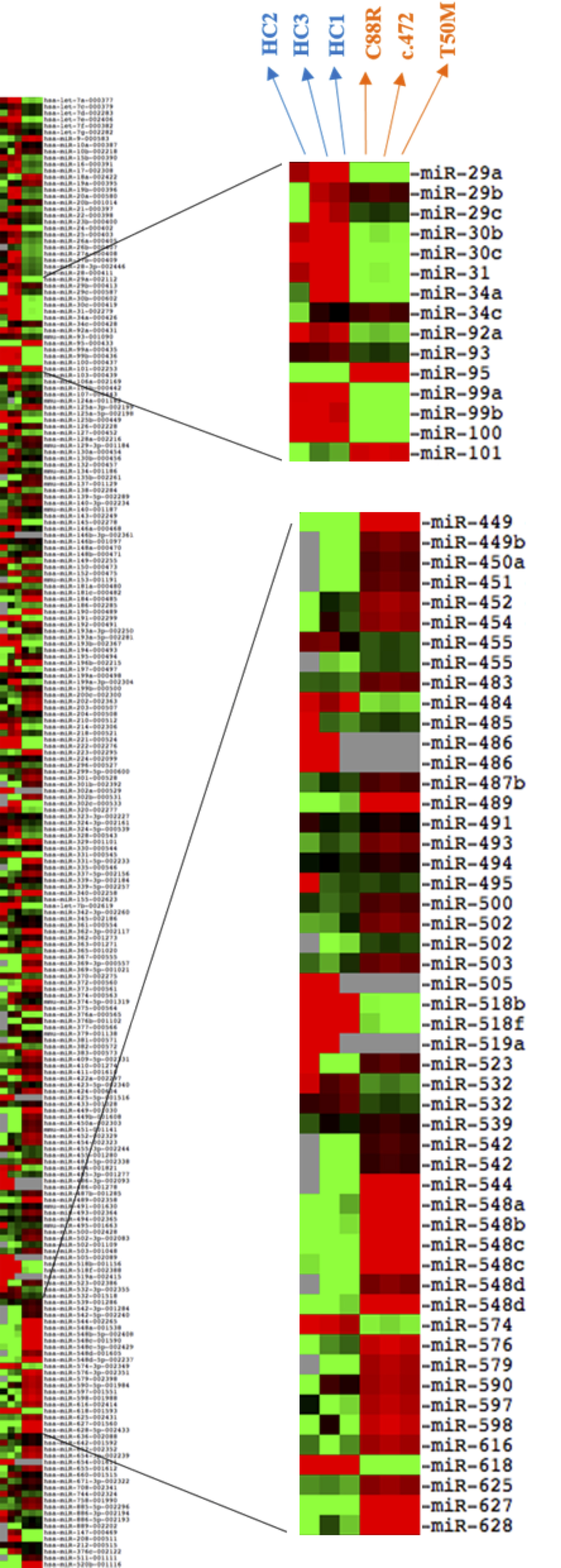

Supplement: Figure S1 — Interleukin (IL)-1β secretion after graded lipopolysaccharide challenge in dermal fibroblast (DF) from healthy control (HC) and patients with tumor necrosis factor (TNF) receptor-associated periodic fever syndrome (TRAPS). Levels of IL-1β secretion observed from TRAPS DF (n = 3) compared with HC (n = 3). The two-way ANOVA (p = ≤ 0.05) test was used to determine statistical significance. [file image_1.jpeg]

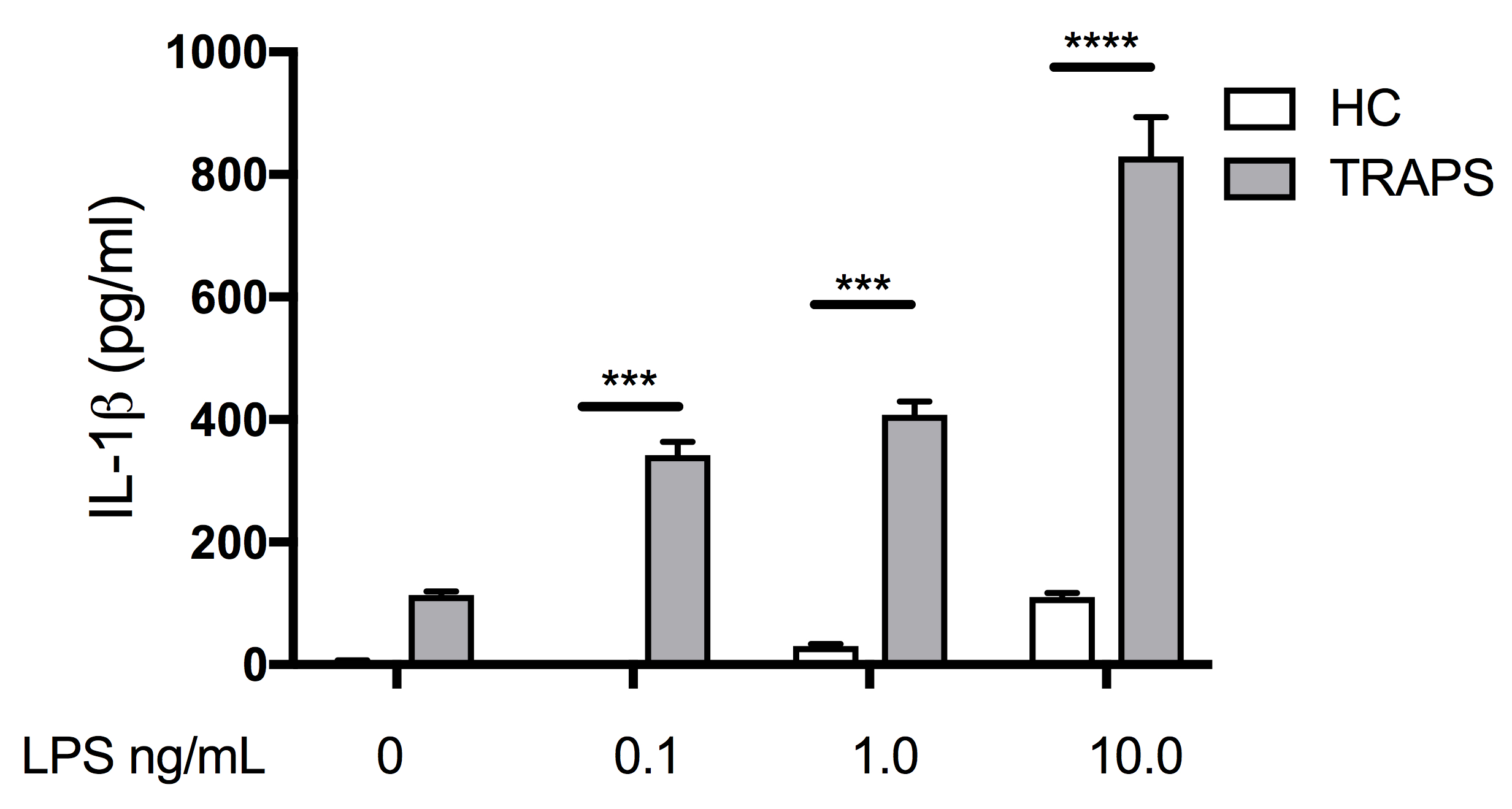

Supplement: Figure S2 — Global expression of microRNAs (miRNAs) in healthy control (HC) and tumor necrosis factor (TNF) receptor-associated periodic fever syndrome (TRAPS). Expression levels of selected miRs observed in TRAPS dermal fibroblast (n = 3) compared with HC (n = 3). Red indicates higher levels of expression, and green indicates lower levels of expression. A section of the cluster analysis has been magnified to illustrate areas that were clearly different between HC and TRAPS. [file image_2.jpeg]
