# Supplementary material for: Inositol-Requiring Enzyme 1-Mediated Downregulation of MicroRNA (miR)-146a and miR-155 in Primary Dermal Fibroblasts across Three TNFRSF1A Mutations Results in Hyperresponsiveness to Lipopolysaccharide
Source: Front Immunol. 2018 Feb 6;9:173. doi: 10.3389/fimmu.2018.00173 (PMC5808292; doi:10.3389/fimmu.2018.00173)
Supplement: Supplementary file 3 [file table_1.doc]

**Table S1. Biological pathways associated with the most significantly differentially expressed**

miRs between unstimulated TRAPS and HC DF (p<0.05)

| **KEGG pathways the miR are predicted to target** | **miRs** | | | | | | | | | |
| --- | --- | --- | --- | --- | --- | --- | --- | --- | --- | --- |
|  | **107** | **204-5p** | **324-3p** | **455-3p** | **483-5p** | **518f-3p** | **548b-5p** | **548c-5p** | **548d-5p** | **598** |
| Ubiquitin mediated proteolysis | X |  |  |  | x |  | X | x | X |  |
| TGF-beta signaling pathway |  |  |  | x |  |  | x | x | X |  |
| mRNA surveillance pathway |  |  |  |  |  |  | x | x | x | X |
| Protein processing in endoplasmic reticulum |  |  |  |  | X |  | x | x | X |  |
| MAPK signaling pathway |  |  |  |  |  |  |  | x | X |  |
| Transcriptional misregulation in cancer |  | X |  | X |  | x | x | x | x | X |
| Fatty acid biosynthesis | X |  |  |  |  |  |  |  |  |  |
| Circadian rhythm | X |  |  |  |  |  | x | x | X |  |
| Hepatitis B | x |  |  |  |  |  | x | x | X |  |
| Hepatitis C |  |  |  |  |  |  | x | x | x | X |
| Pancreatic Cancer | x |  |  |  |  |  | x | x | x |  |
| Wnt signalling pathway | x |  |  |  |  |  | x | x | X |  |
| Drug metabolism – Cytochrome P450 |  |  |  | x |  |  | X | x | X |  |
| Lysine degradation |  |  | X | X |  |  |  |  |  |  |
| Hedgehog signaling pathway | X | X |  |  |  |  | x | x | X |  |
| Glycosaminoglycan biosynthesis - heparan sulfate / heparin |  |  | x |  |  |  | x | x | X |  |
| Notch signaling pathway |  |  |  | X |  |  | x | x | x | X |
| Neurotrophin signaling pathway | X | X |  |  |  |  | x | x | x |  |
| Basal cell carcinoma | X |  |  |  |  |  |  | x | X |  |
| Pathways in cancer | X |  |  |  |  |  |  | x | X |  |
| HTLV-I infection | X |  |  |  |  |  | x | X |  |  |
| N-Glycan biosynthesis |  |  |  |  | X |  |  |  |  |  |
| mTOR signaling pathway | X |  |  |  |  |  |  | X |  |  |
| Cell cycle | X |  |  | X |  |  | X |  |  |  |
| Glycosaminoglycan biosynthesis - chondroitin sulfate |  |  | x |  |  |  | x | x | X |  |
| Prion Disease |  |  |  |  |  |  | x | x | x |  |
| Adherens Junction |  |  |  |  |  |  | x | X | x |  |
| Adipocytokine signaling pathway |  |  |  |  |  |  | x | x | X |  |
| Endocytosis |  |  |  |  |  |  | x | x | X |  |
| Endocrine and other factor-regulated calcium reabsorption |  |  |  |  |  |  | x | x | X |  |
| Epstein-Barr virus infection |  |  |  |  |  |  | x | x | x |  |
